# Gas chromatography-stable isotope ratio mass spectrometry prior solid phase microextraction and gas chromatography-tandem mass spectrometry: development and optimization of analytical methods to analyse garlic (*Allium sativum* L.) volatile fraction

**DOI:** 10.1016/j.heliyon.2024.e30248

**Published:** 2024-04-26

**Authors:** Silvia Pianezze, Mauro Paolini, Angelo Antonio D'Archivio, Matteo Perini

**Affiliations:** a Centro Trasferimento Tecnologico, Fondazione Edmund Mach, Via E. Mach n.2, 38098, San Michele all’Adige, TN, Italy; b Dipartimento di Scienze Fisiche e Chimiche, Università degli Studi dell’Aquila, Via Vetoio, 67100, Coppito, L'Aquila, Italy

**Keywords:** Garlic, Solid phase microextraction, Isotope ratio mass spectrometry, Carbon isotopic ratio, Gas chromatography

## Abstract

Garlic (*Allium sativum* L.) is not only appreciated for its flavour and taste, but it is also recognized for various health properties. The European Commission, through the attribution of the Protected Designation of Origin (PDO) certification mark, has officially recognized some specific varieties of garlic. To protect not only the commercial value but also the reputation of this appreciated product, effective tools are therefore required. For the first time, a new compound specific isotope analysis method based on carbon stable isotopic ratio measurement of the three major volatile garlic compounds allyl alcohol (AA), diallyl disulphide (DD) and diallyl trisulphide (DT) through head-space solid phase microextraction (HS-SPME) followed by gas chromatography-combustion-isotope ratio mass spectrometry (GC-C-IRMS) was developed. A within-day standard deviation (Sr_within-day_) of 0.3 ‰, 0.4 ‰ and 0.2 ‰ for *δ*(^13^C) and a between-day standard deviation (Sr_between-day_) of 0.8 ‰, 1.0 ‰ and 0.6 ‰ of AA, DT and DD was estimated. For the first time, the ranges of isotopic variability for the three volatile compounds of red garlic from two neighbouring Italian regions (Abruzzo and Lazio) were defined analysing 30 samples. The same dataset was also considered in analysing the percentage composition of the previously mentioned three volatile compounds through HS-SPME followed by gas chromatography-tandem mass spectrometry (GC-MS/MS). The two analytical approaches were combined in this explorative study, aiming to provide potential parameters to discriminate garlic samples based on their geographical origin.

## 1 Introduction

Garlic (*Allium sativum* L.) plant belongs to the *Liliaceae* family, renowned not only for its characteristic aroma but also for the health properties of its bulb according to traditional medicine [1,2] and to several scientific studies [[3], [4], [5]]. Garlic figures as an herbaceous plant 30–80 cm high, typically propagated vegetatively using the cloves from the previous harvest, as the cultivated varieties lost the ability to reproduce by true seeds [6]. Garlic plant structure is characterised by an underground part represented by the bulb, divided in numerous cloves, which look like smaller bulbs covered by reddish casings and containing several compounds commonly used in the phytotherapeutic field [6]. They usually contain sulphur, such as allicin, responsible for garlic typical aroma [7]. This compound is released when the enzyme allinase acts on alliin, a colourless and tasteless compound, which is the main component of fresh garlic. In the intact cell, alliin and the other sulfoxides are confined to the cytoplasm, while its hydrolytic enzyme - allinase - is present in the vacuole only: the destruction of the cellular structure of garlic releases the aforementioned enzyme, which causes the hydrolysis of the sulfoxides and their transformation into disulfides and trisulfides [7]. Several studies highlighted potential uses for allicin and other organosulfur compounds deriving from garlic (e.g., diallyl disulfide and diallyl trisulfide) in the medical field: reduction of atherosclerosis and the formation of fatty deposits [8], normalisation of lipoproteins, decrease in blood pressure [9,10], anti-inflammatory and antioxidant properties [[11], [12], [13]], protection against colon cancer [14].

Due to its health properties and applications in the traditional cuisine, the demand for dried garlic keeps growing. The global dehydrated product market size is estimated to be worth $738 million in 2021 and it is expected to reach a size of $948.1 million by 2028 [15]. Europe largely depends on the import to cover the request for this product. The 400 tons of garlic that Europe produces, mainly in Spain, are destined almost exclusively for the fresh garlic market. The annual Italian garlic production is only 33 tons but some local products, such as Voghiera Garlic and Polesine White Garlic, have been awarded the certification mark of the Protected Designation of Origin (PDO) by the European Commission [16]. To protect these added-value products, it is necessary to develop new analytical techniques effective in characterising specific productions and/or tracing their geographical origin. To classify garlic according to the cultivars and/or the geographical origin, high performance liquid chromatography [17], high resolution magic angle spinning-nuclear magnetic resonance (HRMAS-NMR) spectroscopy [18], Fourier-transform infrared spectroscopy [[18], [19], [20]], high resolution mass spectrometry [21], and electronic nose [22] were previously applied. The elemental profile determined by atomic spectroscopy [[23], [24], [25]], the metabolic profiles investigated using liquid chromatography – mass spectrometry (LC/MS) [26] and the headspace gas chromatography-mass spectrometry (HS-GC-MS) analysis of the volatile organosulfur compound profiles [27] allowed for a good geographical discrimination of garlic cultivated in different areas of the world.

The head-space solid phase micro-extraction (HS-SPME) coupled to gas-chromatography with mass spectrometry (GC-MS) was used by Biancolillo and coworkers to analyse the amount of several sulphurate compounds, including diallyl disulphide and diallyl trisulphide, in fresh garlic coming from three different Italian regions [28].

Stable Isotope Ratio (SIR) analysis has been used in the recent decades for the authentication and traceability of different foodstuffs, such as wine, vegetables, meat and spices [29,30].

The SIR analysis of carbon, nitrogen, sulphur, hydrogen and oxygen, so far carried out on the whole bulk freeze dried sample only, has been successfully applied by some authors to characterise garlic from different geographical areas: China [31,32], Thailand, South Korea and Vietnam [[32], [33], [34]], Argentina [32], Slovenia [35] and Italy [36].

Recently, a new SIR analytical approach called “compound-specific isotope analysis (CSIA)" and based on the measurement of the isotopic ratios of the individual compounds of a product has been successfully applied to discriminate food samples coming from different origins and/or to give information about the food production chain [37]. Boecklen et al. reported that CSIA through GC-C-IRMS (gas chromatography-combustion-isotope ratio mass spectrometry) or LC-IRMS (liquid chromatography-isotope ratio mass spectrometry) usually shows several advantages over bulk analysis carried out through EA-IRMS (elemental analysis-isotope ratio mass spectrometry) [38]. Since the metabolic and physiological factors that influence the isotopic values of a single compound are fewer and often better understood than those that influence the whole bulk sample, interpretation via CSIA requires less assumptions than bulk isotopic values. Furthermore, possible issues related to the contamination of the compound of interest with other interfering molecules are less frequent when the sample matrix is simpler, and the individual compounds can be therefore more efficiently separated.

In this study, for the first time, a HS-SPME coupled with GC-C-IRMS method for the CSIA analysis of carbon isotopic ratio *δ*(^13^C) on three major volatile components (allyl alcohol, diallyl disulphide and diallyl trisulphide) of the yet never investigated freeze-dried garlic powder was developed and tested. The within-day and between-days repeatability of this CSIA method were estimated and a preliminary dataset of thirty samples from four sites located in two different Italian regions (Abruzzo and Lazio) was built. Moreover, this new CSIA analysis was integrated by carrying out the percentage composition of the three volatile compounds through HS-SPME followed by GC-MS/MS and a geographical discrimination of the Italian samples was attempted, on an exploratory basis, by integrating the two techniques.

## 2 Materials and methods

### 2.1 HS-SPME coupled with GC-C-IRMS analysis

To carry out the head-space solid phase micro extraction in this study, the PDMS/CAR/DVB (2 cm, 50/30 m) SPME fiber (SigmaAldrich/Supelco, Bellefonte, USA) was used. As reported by Recinella et al., in a comparison between the PDMS–DVB, CAR–PDMS, PA and PDMS/CAR/DVB fibers, the best efficiency, in parallel with the greater number of compounds detected in garlic powder samples, can be achieved with the latter [39]. The following two parameters were evaluated to optimize the extraction conditions during the sampling phase: temperature of the thermostatic bath (adjusted to 20, 40, 60, 80 °C) during the equilibration and sampling phases, and sampling time (20, 30, 40 min).

An exposure of the fiber to the headspace (HS) containing the garlic powder sample (0.5 g) for 30 min at 80 °C was found to be the condition that allowed a greater extraction capacity of the target compounds allyl alcohol (AA), diallyl disulphide (DD) and diallyl trisulphide (DT), compensating for the lower sensitivity of the IRMS compared to the MS. After sampling, fiber was exposed into the GC inlet at 260 °C for 2 min.

The GC-C-IRMS consisted of a GC oven (Trace GC Ultra) equipped with a TriPlus autosampler, retrofitted to the combustion interface GC/IsoLink, connected to the isotope ratio mass spectrometer (Delta V Advantage, Thermo Scientific, Milan, Italy) and, in parallel, to a single-quadrupole GC–MS (ISQ Thermo Scientific, Milan, Italy). The data were collected in triplicate using the Isodat 2.5 software (Thermo Fisher Scientific). A capillary column ZB-WAX 30 m × 0.32 mm × 0.5 μm (Phenomenex, Torrance, California, United States) was used to separate the single compounds. The carrier gas was He at a constant flow of 2.0 mL/min. The oven temperature was programmed to increase during each run from 50 °C (held for 1 min) to 250 °C at 5 °C/min (held for 6 min). The described oven temperature program was chosen to ensure the high chromatographic resolution required for the IRMS analysis. Injection was performed at 260 °C in splitless mode.

After the GC column, a first splitter is set up to divide the flow into two parts, sending 1/10 to a conventional MS and the remaining 9/10 to the IRMS to determine the *δ*(^13^C). The eluted compounds were combusted into CO_2_ and H_2_O in a combustion furnace reactor (GC/Isolink), operating at 1030 °C and consisting of a non-porous alumina tube (320 mm long) containing three wires (Ni/Cu/Pt, 0.125 mm diameter, all 240 mm long) braided and centred end-to-end within the tube. A Nafion® membrane inside a water trap removes water vapor from the eluent/sample. The He carrier gas and CO_2_ reference gas were set respectively at 1 and 0.6 bar. The Faraday collector was set to detect CO_2_ ion contributions at *m*/*z* 44, 45 and 46. The MS was represented by a single-quadrupole (ISQ Thermo Scientific, Bremen) having the aim to identify the compounds exiting the GC column. The sample was carried from the GC to the MS through a transfer line, set at a temperature of 250 °C. The temperature of the MS ion source was set at 250 °C. The GC-MS analyses were performed in the full scan mode (35–600 *m*/*z*).

Among the compounds detected, allyl alcohol, diallyl disulfide and diallyl trisulfide presented the highest relative concentration, giving well-resolved peaks, and were therefore chosen as target.

The NIST library (NIST Standard Reference Database 1A NIST/EPA/NIH Mass Spectral Library (NIST 08) and NIST Mass Spectral Search Program (Version 2.0f)) was used to identify the different compounds (through comparison between the mass spectra acquired at 70 eV with library data) and confirmed by observing the retention time by injecting the three pure compounds.

### 2.2 Expression and correction of stable isotope ratio analysis data and data correction

According to the IUPAC protocol [40], the ^13^C/^12^C values are expressed in the delta scale (δ‰), against the international standards V-PDB (Vienna-Pee Dee Belemnite) according to Equation (1):

(1) $$\delta_{ref}\left(i_{E}/j_{E},sample\right)=\left[\frac{R\left(i_{E}/j_{E},sample\right)}{R\left(i_{E}/j_{E},ref\right)}\right]-1$$

where *ref* is the international measurement standard, sample is the analysed sample, and *i_E_/j_E_* is the isotope ratio between heavier and lighter isotopes. The delta values were multiplied by 1000 and expressed in units “per mil” (‰). Each sample was analysed in triplicate.

The *δ*(^13^C) values were corrected for the instrumental drift and calculated against standard allyl alcohol (−24.8 ± 0.2 ‰), diallyl disulfide (−29.9 ± 0.2 ‰) and diallyl trisulfide (−28.6 ± 0.2 ‰) and injected in triplicate at the beginning and at the end of each analytical sequence. The real values of the working standards were obtained by analysing them through an elemental analyser-isotope ratio mass spectrometry EA-IRMS (Flash 1112, Thermo Scientific, Bremen, Germany) interfaced to a DELTA V isotope ratio mass spectrometer (Thermo Scientific) through a ConFlo IV dilutor (Thermo Finnigan, Bremen, Germany) against the international standards USGS 40 (*δ*(^13^C) = −26.39 ± 0.04 ‰) and IAEA–CH–6 sucrose (*δ*(^13^C) = −10.45 ± 0.04 ‰) (U.S. Geological Survey, Reston, VA, USA), NBS-22 fuel oil (*δ*(^13^C) = −30.03 ± 0.05 ‰), (IAEA-International Atomic Energy Agency, Vienna, Austria).

### 2.3 Percentage composition of the volatile compounds through HS-SPME coupled with GC-MS/MS

The GC-MS/MS analysis was performed on an Agilent Intuvo 9000 gas chromatograph coupled to a 7000C Triple Quadrupole mass spectrometer (Agilent Technologies, Santa Clara, CA). The GC-MS/MS system was equipped with a PAL RSI 85 sampler (Zwingen, Switzerland) for the automated HS-SPME analysis. Agilent MassHunter WorkStation - Qualitative Analysis software (ver. B.08.00) was used for data analysis. Extraction of volatiles was reached using a DVB/CAR/PDMS (1 cm, 50/30 μm) SPME fiber (Supelco, Bellefonte, PA, U.S.A.) that was preconditioned according to the manufacturer's instructions (250 °C for 30 min). The choice of the fiber, as explained in section 2.1, was made based on the suggestions of Recinella et al. [39].

Based on the method optimization study (see section 2.1), the best working condition for the extraction of all the volatile compounds consisted in placing 0.5 g of garlic samples into a 20 mL glass vial with a Teflon-coated septum, incubating the sample at 40 °C, agitating it at 250 rpm for 5 min and extracting it at 40 °C for 20 min. The fiber was desorbed for 10 min at 250 °C in the injection port (Multimode Inlet System, Agilent) operated in split mode (1:5) equipped with a 0.75 mm i.d. Ultra Inert SPME Liner (Agilent).

The GC analysis was carried out on a DB‐Wax Ultra Inert (20 m × 0.18 mm × 0.18 μm; Agilent Technologies, Inc.). The oven temperature was programmed starting at 40 °C for 2 min, raised to 55 °C by 10 °C/min, then raised to 165 °C by 20 °C/min, and finally raised to 240 °C by 40 °C/min and held at this temperature for 5 min. He as carrier gas at a flow of 0.8 mL/min. The MS transfer line temperature was set to 250 °C. Ionization was performed in electron impact (EI) mode at 70 eV, ion source and MS quadrupole temperatures were 230 °C and 150 °C. These were the best conditions for a fast chromatography as reported by Khatri et al. [41]. The mass spectra were acquired in full scan mode from 33 to 400 *m*/*z*. Detected peaks were identified using NIST library (Version 2.4) search comparing their fragment pattern and the composition of the sample was expressed using the relative percentage of volatile compounds. A total number of 46 volatile compounds were identified and the relative percentage of each was calculated as a percentage of the total volatile fraction (%TVF) (see Supplementary Table 1S).

### 2.4 Explorative study of traceability: samples description and preparation

**Fig. 1:**
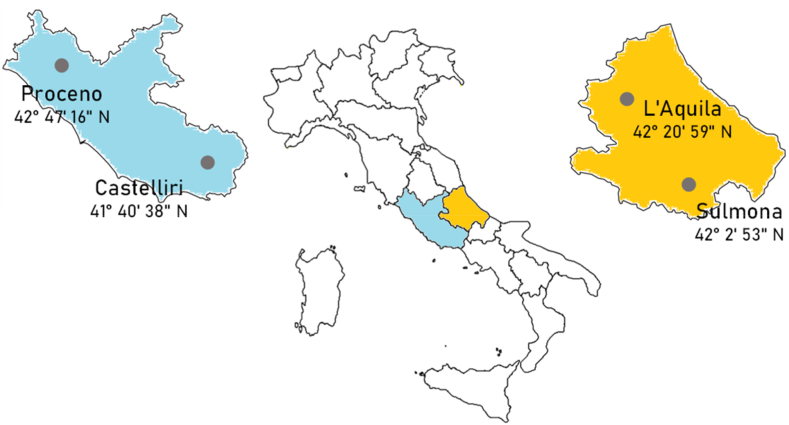
Representation of the sampling points together with their latitudes: Sulmona and L'Aquila in Abruzzo; Proceno and Castelliri in Lazio.

In this study, 30 samples of garlic coming from the territories of Sulmona (n = 9), L'Aquila (n = 4) in the Italian Abruzzo region, and Proceno (n = 12) and Castelliri (n = 5) from Lazio region (Fig. 1) were analysed. Garlic samples were acquired from the consortia working in the previously mentioned territories or from local producers who assessed and guaranteed their authenticity. Within the individual consortia, common agronomic practices used to be shared in the production of garlic bulbs which should guarantee a certain homogeneity of the garlic varieties produced in the different areas.

Three cloves were taken from each garlic bulb, separated from the peel and cut into pieces, obtaining approximately 5 g of product. The samples thus prepared were lyophilized with a 5PASCAL (Trezzano sul Naviglio MI, Italy) freeze dryer model LIO5P DIGITAL during 24h at a temperature of −50 °C and a pressure of 7*10^−1^ mbar and ground in a steel mortar for 10 min to obtain a powder. The samples were left at a temperature of approximately 23 °C for three days to allow complete degradation of the allicin (see section 3.1).

### 2.5 Statistical analysis

Statistica For Windows v 13.1 (StatSoft Inc., Tulsa, OK, USA) was used as data evaluation software and significance differences were found by applying the Tukey HSD test. In all cases, the cutoff value was set at p < 0.05, which is associated with a significant difference between groups of values.

Through the application of principal component analysis (PCA), the major variance sources of the stable isotope ratio data matrix were described. Patterns in a data matrix can be emphasized by projecting objects and variables into the space of a few significant PCs with minimal information loss.

## 3 Results and discussion

### 3.1 Selection of the volatile component for the analyses

An odourless derivative of cysteine, the alliin [S-3-(2-propenylsulfinyl)-l-alanine-], is contained in fresh garlic. The enzyme alliinase, when a fresh garlic bulb is crushed, can convert alliin to allicin (S-allyl 2-propene-1-sulfinothioate), which is responsible for crushed fresh garlic characteristic odour [42]. Unfortunately, allicin is a highly unstable molecule and it degrades in 16 h at 23 °C [43,44] producing a wide variety of organosulfur compounds. The SIR analysis of this volatile component of crushed fresh garlic should therefore be avoided. Volatilization, diffusion and absorption are physical processes that are associated with isotopic effects that cannot be neglected [45] as the variations in the isotope ratio recorded could be due to fractionation processes taking place on the molecule during its degradation rather than to geographical effects.

For this reason, in this study, we focused on the final products deriving from the complete degradation of allicin, a volatile component of ground and freeze-dried garlic (see section 2.4), which can be considered stable over time (and therefore not subject to fractionation). Specifically, allyl alcohol (AA), diallyl trisulphide (DT) and diallyl disulphide (DD) were analysed as they represented the most abundant compounds in terms of relative concentration. As reported by Abe et al., they could be found in greater quantities in aged than in fresh garlic extract [46] and could therefore be more easily analysed with the GC-C-IRMS, usually requiring larger sample amounts than the classic GC-MS quantification [41]. Moreover, the matrix we considered (freeze-dried garlic powder) can be easily obtained by fresh bulbs and it represents a common commercial product available on the market. This results in the possibility to expand the applications of the method.

### 3.2 Validation of the HS-SPME coupled with GC-C-IRMS method for CSIA analysis

To test the within-day repeatability of the method, the same sample was analysed ten times in a row and the mean and standard deviation were calculated. The results are reported in Table 1. By applying the tests Shapiro Wilks 5 % and Huber 5 % to the data, normal distribution and absence of outlier were found. A within-day standard deviation (Sr_within-day_) of 0.3 ‰, 0.4 ‰ and 0.2 ‰ for *δ*(^13^C) of the different volatile compounds AA, DT and DD was calculated. This is in line with the within-day standard deviations estimated for the analysis of other volatile compounds analysed through GC-C-IRMS technique, such as vanillin [47].

**Table 1:** Within-day repeatability and between-day repeatability of the CSIA method. The mean *δ*(^13^C) for AA, DT and DD are reported together with the within-day and between-day Sr (‰). The^13^C/^12^C values are expressed in the delta scale (δ‰), against the international standards V-PDB (Vienna-Pee Dee Belemnite).

	Allyl alcohol (AA) *δ*(^13^C)(vs V-PDB‰)	Diallyl Trisulphide (DT) *δ*(^13^C)(vs V-PDB‰)	Diallyl disulphide (DD) *δ*(^13^C)(vs V-PDB‰)
**1**	−47.1	−38.0	−35.1
**2**	−46.9	−38.1	−35.0
**3**	−47.4	−37.4	−35.1
**4**	−47.5	−37.6	−35.1
**5**	−47.5	−38.3	−35.2
**6**	−47.7	−38.4	−35.5
**7**	−47.9	−38.4	−35.4
**8**	−47.4	−37.8	−35.4
**9**	−47.5	−37.8	−35.1
**10**	−47.4	−38.4	−35.0
**mean**	**−47.4**	**−38.0**	**−35.2**
**st.dev.**	**0.3**	**0.4**	**0.2**
	Allyl alcohol (AA) *δ*(^13^C) (vs V-PDB‰)	Diallyl Trisulphide (DT) *δ*(^13^C) (vs V-PDB‰)	Diallyl disulphide (DD) *δ*(^13^C) (vs V-PDB‰)
**Day 1**	−43.7	−34.5	−31.0
**Day 2**	−42.9	−32.5	−32.0
**Day 3**	−44.4	−33.0	−32.1
**mean**	**−43.7**	**−33.3**	**−31.7**
**st.dev.**	**0.8**	**1.0**	**0.6**

A different sample was considered to estimate the between-day standard deviation and check whether measurements in different days could lead to significant differences in the *δ*(^13^C) of the three volatile compounds considered. Therefore, the same sample has been measured in three consecutive days. The results are reported in Table 1. A between-day standard deviation (Sr_between-day_) of 0.8 ‰, 1.0 ‰ and 0.6 ‰ for *δ*(^13^C) of the different volatile compounds AA, DT and DD was calculated.

### 3.3 First explorative application of the HS-SPME coupled with GC-C-IRMS method in the geographical discrimination of garlic

**Fig. 2:**
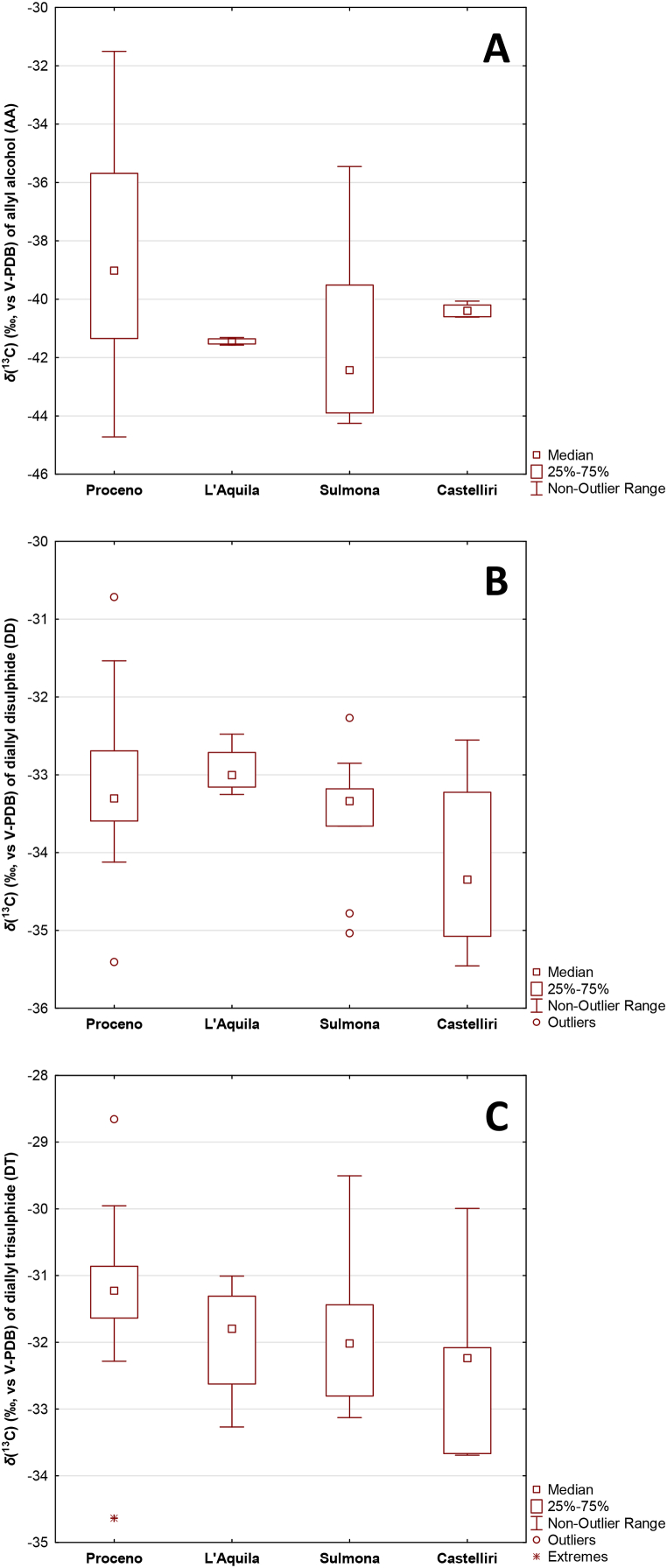
Carbon isotopic ratios *δ*(^13^C) of garlic volatile compounds: **A** allyl alcohol (AA), **B** diallyl disulphide (DD) and **C** diallyl trisulphide (DT). No statistical differences were detected (p > 0.05).

As a first application of the HS-SPME coupled with GC-C-IRMS method to obtain a preliminary dataset, samples coming from two different Italian regions have been considered (Fig. 2).

Due to geographical proximity, the samples were ordered according to geographical coordinates (latitude) rather than by regions of provenance (from north to south: Proceno 42° 47′ 16″ N, L'Aquila 42° 20′ 59″ N, Sulmona 42° 2′ 53″ N and Castelliri 41° 40′ 38″ N). As reported in Fig. 2C, based on DT *δ*(^13^C), it is possible to observe a discrimination among the four groups with lower values for the northern samples of Proceno (mean −31.29 ‰), followed by L'Aquila and Sulmona (mean −31.96 ‰) and finally the southern samples of Castelliri (mean −32.33 ‰). These differences, although not statistically significant (p > 0.05), are more evident when looking at DT *δ*(^13^C) rather than at DD *δ*(^13^C) (mean from −33.10 ‰ for Proceno and −34.13 ‰ for Castelliri) (Fig. 2B), while they are irrelevant when considering AA *δ*(^13^C) (Fig. 2A), which is the least discriminating parameter [48].

The AA in garlic plants can be synthesised by following two different pathways: either by the self-condensation reaction of allicin or by the reaction between alliin, the precursor of allicin, and water through a thermal degradation [43]. The two biosynthetic ways, being temperature dependent, could induce a different and potentially significant isotopic fractionation in the conversion of the precursor into allyl alcohol. This could be the reason for a higher variability of the *δ*(^13^C) of this compound and the consequent lower discriminating effectiveness.

### 3.4 Study of the volatile fraction

Some authors investigated the potential of the relative content of garlic volatile compounds as a technique for the geographical traceability of this product. Mi et al. reported that DD can discriminate among garlic samples from different Chinese regions (Dali region vs others) [24]. For Biancolillo et al., DD and DT are parameters able to significantly discriminate among different Italian garlic samples [28].

**Fig. 3:**
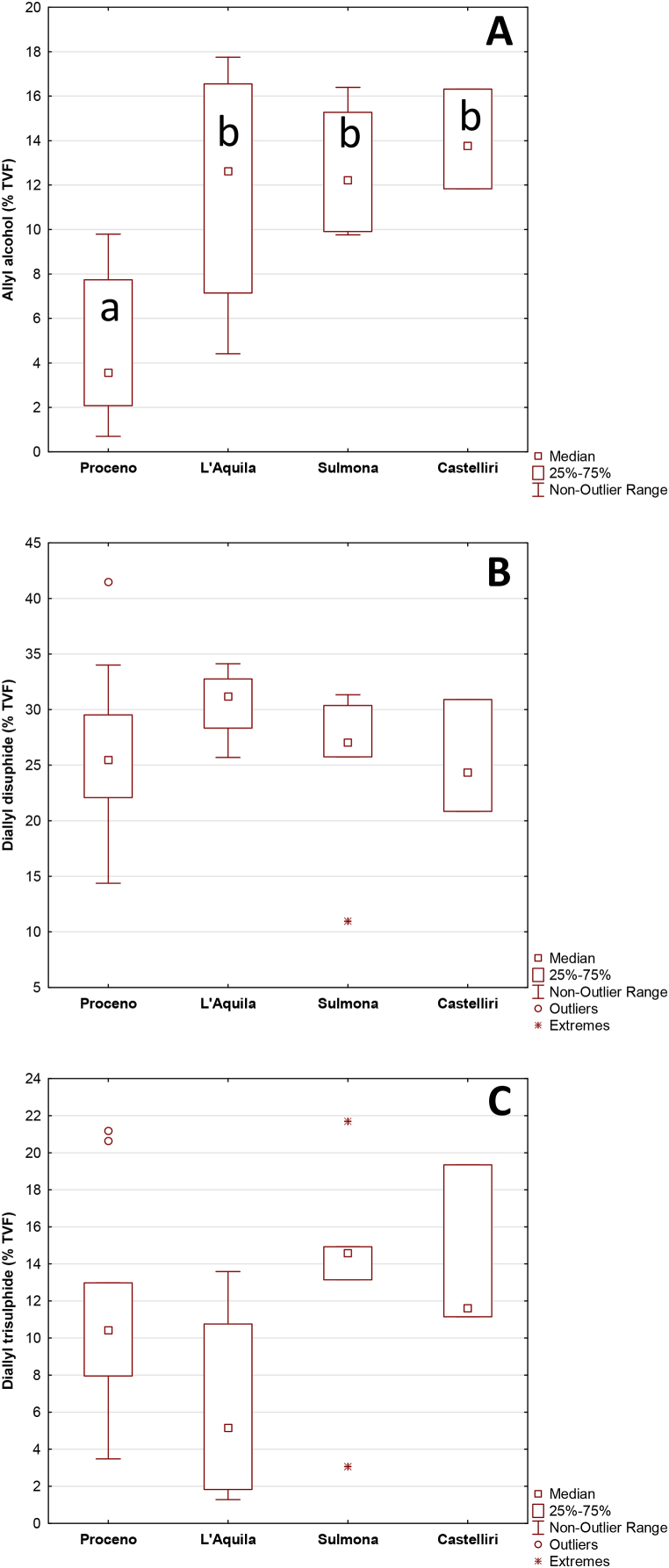
Relative amounts (expressed as % of the total volatile fraction, TVF) of garlic volatile compounds: **A** allyl alcohol (AA), **B** diallyl disulphide (DD) and **C** diallyl trisulphide (DT). No statistical differences were detected (p > 0.05) for DD and DT. Significantly different values (Kruskal-Wallis, p < 0.05) between groups are indicated with different letters for AA.

The relative amounts of the three investigated volatile compounds are shown in Fig. 3. Samples from the northernmost location in the dataset (Proceno) showed significantly higher values of AA (Fig. 3A) respect to the others (p < 0.05). The contents of DD (Fig. 3B) and DT (Fig. 3C) are not significantly different (p > 0.05) between the geographical origins. As previously commented, the lack of difference could be mainly attributed to the geographical proximity of the sampling areas.

### 3.5 Multivariate statistical analysis

A multivariate statistical analysis was performed by PCA exploration of the matrix obtained by combination of stable isotope data and relative gas-chromatographic peak areas of the three target volatiles. A limited number of samples (three from Sulmona, two from Proceno and two from Castelliri), characterised by CISA but not by GC-MS/MS, were excluded from PCA exploration.

**Fig. 4:**
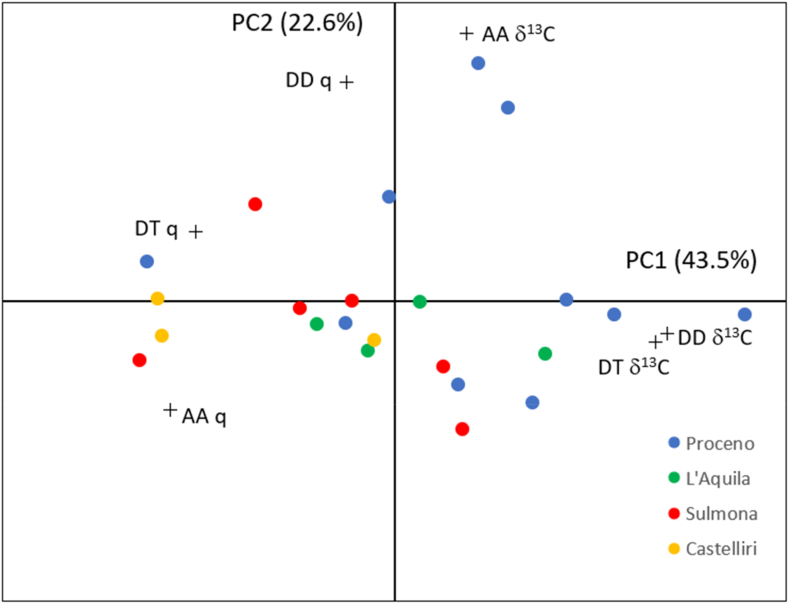
Garlic samples and variables projected onto the plane of the first two Principal Components extracted from the matrix combining carbon isotopic ratios and relative gas-chromatographic areas of allyl alcohol (AA), diallyl trisulphide (DT) and diallyl disulphide (DD).

Each of the four categories can be assumed to be homogeneous based on the geographical origin of garlic samples which are obtained by propagation of local bulb varieties in each of the investigated sites. The PCA results are summarised in Fig. 4, which displays the variables and objects simultaneously projected into the space of the first four PCs, explaining together 66.1 % of the total variance.

The biplot does not reveal a clear separation of garlic samples according to the geographical origin and the relatively large intra-class variability of garlic samples cultivated in the Proceno area seems to be the dominant effect. However, there is a tendency for samples belonging to the same territory to group together. In this context, the geographical proximity of the sampling sites and the low number of samples should be considered. It is worth noting that the samples from the two better represented sites (Proceno and Sulmona) are separated, although not completely, along the bisector of the PC1-PC2 plane. Moreover, it has to be noticed that all six variables, contributing with non-negligible loadings to one or both PCs, seems to have a significant role in the discrimination of garlic samples.

For a more thorough verification of the potential of the three considered organic compounds in the discrimination between different regions, the number of samples should be increased. Also, garlic samples belonging to foreign competitors should be considered to look for possible differences in the *δ*(^13^C) of AA, DT and DD.

## 4 Conclusions

An isotopic method (HS-SPME coupled with GC-C-IRMS) to analyse the *δ*(^13^C) of three volatile compounds, allyl alcohol (AA), diallyl trisulphide (DT) and diallyl disulphide (DD), in powder lyophilized garlic samples was developed. The estimated within-day and between-day repeatability is comparable with that found for other matrices and for similar CSIA methods. The investigated volatile molecules turned out to have the highest relative concentration in garlic powder after freeze-drying. Being degradation products of allicin, they are more easily measured with the HS-SPME coupled with GC-C-IRMS.

A first approach of this technique for the geographical traceability of garlic samples from two Italian neighbouring regions (Abruzzo and Lazio) was provided. Although not statistically significant, a trend in the average *δ*(^13^C) of both AA, DD and DT for the different considered areas could be detected. Indeed, higher *δ*(^13^C) of the three parameters corresponded to samples coming from Proceno, characterised by higher latitude than the other sampling sites. On the other hand, decreasing *δ*(^13^C) values corresponded to samples coming from L'Aquila, Sulmona and Castelliri, characterised by progressively lower latitudes. Furthermore, the volatile fraction of garlic powder was studied through HS-SPME-GC-MS/MS to quantify the previously mentioned compounds. The northernmost location in the dataset (Proceno) showed relatively high percentage of AA with respect to the others, while the DD and DT content is not significantly different between the samples of the two geographical origins.

To test the origin of commercial garlic samples using these two analytical approaches, it will be necessary to delve deeper into this first exploratory study by analysing a wider dataset (including Italian and foreign samples) and considering more volatile compounds. This promising technique, eventually combined with other analytical approaches, could provide a valid answer to the need for garlic powder authentication.

## Data availability

The data that has been used is confidential.

## CRediT authorship contribution statement

**Silvia Pianezze:** Writing – original draft, Investigation, Formal analysis, Data curation, Conceptualization. **Mauro Paolini:** Validation, Investigation, Formal analysis. **Angelo Antonio D'Archivio:** Writing – original draft, Data curation. **Matteo Perini:** Writing – review & editing, Validation, Supervision, Project administration, Methodology, Investigation, Data curation.

## Declaration of competing interest

The authors declare that they have no known competing financial interests or personal relationships that could have appeared to influence the work reported in this paper.
