# Supplementary material for: Gas chromatography-stable isotope ratio mass spectrometry prior solid phase microextraction and gas chromatography-tandem mass spectrometry: development and optimization of analytical methods to analyse garlic (Allium sativum L.) volatile fraction
Source: Heliyon. 2024 Apr 26;10(9):e30248. doi: 10.1016/j.heliyon.2024.e30248 (PMC11078878; doi:10.1016/j.heliyon.2024.e30248)
Supplement: Multimedia component 1 [file mmc1.docx]

Table 1S. Relative percentage (%) of volatile compounds in garlic sample.

| **Compound** | **min** | **max** | **mean** | **median** |
| --- | --- | --- | --- | --- |
| propene | 0.18 | 0.85 | 0.33 | 0.31 |
| alanine | 0.00 | 0.48 | 0.17 | 0.14 |
| dimethyl sulfide | 0.00 | 1.49 | 0.21 | 0.08 |
| isopropyl alcohol | 0.00 | 3.71 | 0.82 | 0.65 |
| sulfide allyl methyl | 0.00 | 3.40 | 0.40 | 0.27 |
| 2-butenal | 0.00 | 0.97 | 0.35 | 0.29 |
| dimethyl disulfide | 0.00 | 1.38 | 0.23 | 0.15 |
| hexanal | 0.00 | 7.45 | 1.08 | 0.17 |
| 2-butanal. 2-methyl | 0.00 | 0.67 | 0.32 | 0.30 |
| allyl alcohol | 0.70 | 17.75 | 8.74 | 7.98 |
| ethylbenzene | 0.00 | 10.67 | 1.00 | 0.29 |
| p-xilene | 0.00 | 5.36 | 0.50 | 0.14 |
| diallyl sulfide | 0.47 | 8.15 | 2.47 | 2.15 |
| 2-pentene. 1-butoxy | 0.00 | 2.67 | 0.64 | 0.36 |
| 4-heptanal | 0.00 | 2.88 | 1.02 | 0.93 |
| o-xilene | 0.00 | 1.70 | 0.15 | 0.00 |
| 1-butanol. 3-methyl | 0.00 | 21.95 | 2.64 | 1.25 |
| (E.E)-2.4-nonadienal | 0.00 | 0.60 | 0.10 | 0.06 |
| 2-methylenecyclohexanol | 0.00 | 1.84 | 0.34 | 0.24 |
| (E)-2-tridecen-1-ol | 0.00 | 3.44 | 0.47 | 0.23 |
| (E)-1-methyl-2-(prop-1-en-1-yl)disulfane | 0.00 | 0.33 | 0.11 | 0.11 |
| disulfide. methyl 1-propenyl | 1.54 | 9.78 | 4.94 | 4.34 |
| (Z)-1-methyl-2-(prop-1-en-1-yl)disulfane | 0.00 | 1.00 | 0.31 | 0.26 |
| pyridine. 3-methyl- | 0.00 | 0.53 | 0.24 | 0.26 |
| pyridine. 2.5-dimethyl | 0.00 | 0.43 | 0.10 | 0.08 |
| 2-hexanal. 2-ethyl- | 0.00 | 4.22 | 1.08 | 0.75 |
| 1-octene. 2-methyl- | 0.00 | 0.96 | 0.11 | 0.05 |
| cyclohexanol. 1-butyl- | 0.00 | 1.61 | 0.52 | 0.45 |
| dimethyl trisulfide | 0.00 | 3.56 | 1.17 | 0.90 |
| 2.6-heptadienal. 2.4-dimethyl | 0.00 | 3.35 | 0.98 | 0.81 |
| pyridine. 3-ethyl-4-methyl | 0.00 | 0.74 | 0.15 | 0.12 |
| acetic acid | 0.20 | 8.21 | 1.72 | 1.43 |
| (E)-1-allyl-2-(prop-1-en-1-yl)disulfane | 0.00 | 1.76 | 0.86 | 0.90 |
| diallyl disulphide | 10.88 | 41.73 | 28.85 | 29.52 |
| (Z)-1-allyl-2-(prop-1-en-1-yl)disulfane | 0.80 | 4.74 | 2.31 | 2.22 |
| 3H-1.2-dithiole | 0.00 | 1.77 | 0.67 | 0.60 |
| 2-octenal. 2-butyl | 0.25 | 2.48 | 0.80 | 0.56 |
| 2.6-octadienal. 3.7-dimethyl-. (E)- | 0.00 | 0.86 | 0.17 | 0.14 |
| trisulfide. methyl 2-propenyl | 1.07 | 22.35 | 11.81 | 12.59 |
| 1-(2-methyl-3-(methylthio)allyl)cyclohex-2-enol | 0.38 | 4.59 | 1.40 | 0.98 |
| bis(1.3-dimethylbutyl)methylphosponate | 0.31 | 3.70 | 1.38 | 0.97 |
| 3-vinyl-1.2-dithiacyclohex-4-ene | 0.46 | 15.46 | 2.53 | 1.55 |
| diallyl trisulphide | 1.28 | 23.40 | 11.84 | 12.65 |
| hexanoic acid | 0.00 | 8.18 | 1.30 | 0.19 |
| 2-vinyl-4H-1.3-dithiine | 0.33 | 10.45 | 2.55 | 1.88 |
| 1-((E)-prop-1-en-1-yl)-2-(Z)-prop-1-en-1-yl)disulfane | 0.00 | 0.79 | 0.12 | 0.00 |
